# Prioritizing flexible working memory representations through retrospective attentional strengthening

**DOI:** 10.1016/j.neuroimage.2023.119902

**Published:** 2023-01-25

**Authors:** Dongwei Li, Yiqing Hu, Mengdi Qi, Chenguang Zhao, Ole Jensen, Jing Huang, Yan Song

**Affiliations:** a State Key Laboratory of Cognitive Neuroscience and Learning & IDG/ McGovern Institute for Brain Research, Beijing Normal University, Beijing, China; b Centre for Human Brain Health, University of Birmingham, Birmingham, UK; c Center for Collaboration and Innovation in Brain and Learning Sciences, Beijing Normal University, Beijing, China; d Center for Cognition and Neuroergonomics, State Key Laboratory of Cognitive Neuroscience and Learning, Beijing Normal University, Zhuhai, China

**Keywords:** Working memory, Attention, EEG, Multivariate pattern analysis, Frontoparietal network, Phase-amplitude coupling

## Abstract

Previous work has proposed two potential benefits of retrospective attention on working memory (WM): target strengthening and non-target inhibition. It remains unknown which hypothesis contributes to the improved WM performance, yet the neural mechanisms responsible for this attentional benefit are unclear. Here, we recorded electroencephalography (EEG) signals while 33 participants performed a retrospective-cue WM task. Multivariate pattern classification analysis revealed that only representations of target features were enhanced by valid retrospective attention during retention, supporting the target strengthening hypothesis. Further univariate analysis found that mid-frontal theta inter-trial phase coherence (ITPC) and ERP components were modulated by valid retrospective attention and correlated with individual differences and moment-to-moment fluctuations on behavioral outcomes, suggesting that both trait- and state-level variability in attentional preparatory processes influence goal-directed behavior. Furthermore, task-irrelevant target spatial location could be decoded from EEG signals, indicating that enhanced spatial binding of target representation is vital to high WM precision. Importantly, frontoparietal theta-alpha phase-amplitude coupling was increased by valid retrospective attention and predicted the reduced random guessing rates. This long-range connection supported top-down information flow in the engagement of frontoparietal networks, which might organize attentional states to integrate target features. Altogether, these results provide neurophysiological bases that retrospective attention improves WM precision by enhancing flexible target representation and emphasize the critical role of the frontoparietal attentional network in the control of WM representations.

## 1 Introduction

The cognitive resources of working memory (WM) are limited but flexible, in which attention acts as a gate, selectively allowing task-relevant information to enter WM, thereby supporting upcoming behaviors (Gazzaley and Nobre, 2012). Although rich evidence supports attentional promotion on WM representations, the underlying cognitive processing and neural substrates remain unclear. Additionally, WM plays a fundamental role in fluid intelligence and broad cognitive function (Conway et al., 2003; Unsworth et al., 2014). Studying how attention benefits WM provides new insight into training individuals with memory deficits.

Previous work proposed two distinct hypotheses of attentional benefits on WM: target strengthening (Souza et al., 2015) and non-target inhibition (Oberauer, 2001). Several studies demonstrated the costs of invalid anticipatory attention (Gunseli et al., 2015; Griffin and Nobre, 2003), and larger behavioral benefits were observed as more non-targets were removed from WM with increasing memory loads (Van Moorselaar et al., 2015). However, recent behavioral studies claimed that attending to the cued object in WM leads to stronger binding of that object to its context without affecting the strength of non-cued objects (Rerko and Oberauer, 2013). Several fMRI studies also found support for enhanced WM representations of the cued object (Lepsien and Nobre, 2007; Lewis-Peacock et al., 2012; Sprague et al., 2016). Although previous EEG studies have examined the ERP of retro-cue benefits (Lepsien and Nobre, 2006), ERP responses cannot be assigned to directly represent content held in WM and few studies have examined both target and non-target representations held in WM simultaneously. There is still a lack of direct neurophysiological findings examining both target and non-target representations in relation to the underlying oscillatory mechanisms supporting one of the two distinct hypotheses of attention.

One way to examine the attentional effect on WM is to adopt retrospective cues (retro-cue) under WM tasks (van Ede et al., 2019a; van Ede and Nobre, 2023). The pre-cue WM task has been widely used previously (Panichello and Buschman, 2021; Vogel and Machizawa, 2004; Zhao et al., 2022a). In pre-cue tasks, cues are presented before the encoding period and reduce the memory load, confusing the retrieval of pure attentional effects. However, in retro-cue tasks, retro-cues are presented during retention and obtain cleaner attentional effects, which involves top-down executive control on WM representations. The frontal N2 has been well-investigated in executive control tasks (Hong et al., 2021; Ullsperger et al., 2014). One core component of executive functions is to select task-relevant information and ignore task-irrelevant competing sources according to the current goal. Previous studies found that retro-cues could modulate attention during retention (Kuo et al., 2012) and prioritize relevant representations (Sahan et al., 2020). However, the frontal N2 has not been examined and linked to behavioral performance in retrospective WM tasks.

Attention-related modulatory signals interact with mnemonic representations (de Vries et al., 2020), indicating the flexibility of information held in WM. The posterior alpha activity (8–12 Hz) has been found to be related to attentional modulations and may be associated with inhibition in the early visual cortex (Gutteling et al., 2022; Jensen and Mazaheri, 2010). However, the role of alpha oscillations in the inhibitory hypothesis has been challenged by findings from spatial attention and WM tasks (Schroeder et al., 2018; Foster and Awh, 2019), which supports the idea that alpha oscillations rather are involved in target enhancement and feature integration (Ghiani et al., 2021; Parto Dezfouli et al., 2021; Zhang et al., 2019). Recent studies showed that the frequency of alpha oscillations would be slower when two stimuli need to be integrated into one object (Sharp et al., 2022; Wutz et al., 2018). In addition, although abundant studies have reported a relationship between visual attention and alpha event-related desynchronization (ERD; Woodman et al., 2021), recent work stated that modulation in alpha amplitudes by retro-cues in posterior areas during retention is an automatic shift of attention (Schneider et al., 2015; Poch et al., 2017), which is not directly involved in mnemonic prioritization (Mössing and Busch, 2020). Therefore, the role of alpha oscillations and how attentional modulation interacts with retrospective WM remains unclear.

Frontal oscillatory signals also play a vital role in the control of WM. Mid-frontal theta activity (3–7 Hz) was related to attentional control (Griesmayr et al., 2010) and played a vital role in goal-related prioritization during WM (de Vries et al., 2020; Riddle et al., 2020). Previous studies found that midline theta power was involved in the control of WM (Hsieh and Ranganath, 2014). Visuospatial attention could modulate the connection within frontoparietal networks. Recent work further found the coherence between frontal and parietal theta oscillations during retention, which supported the theta phase contributions in the modulation of WM representations. Connections between the frontal and parietal cortices were related to top-down information flow (Buschman and Miller, 2007; Marshall et al., 2015). Previous work reported a correlation between frontal theta power and posterior alpha power (Popov et al., 2018) and found a phase-amplitude coupling (PAC) between frontal theta and parietal alpha activities in local field potentials when non-human primates performed spatial attention tasks (Fiebelkorn et al., 2018). As such, the information flow in WM controlled by retrospective attention might be related to the frontal theta parietal alpha connection.

**Fig. 1:**
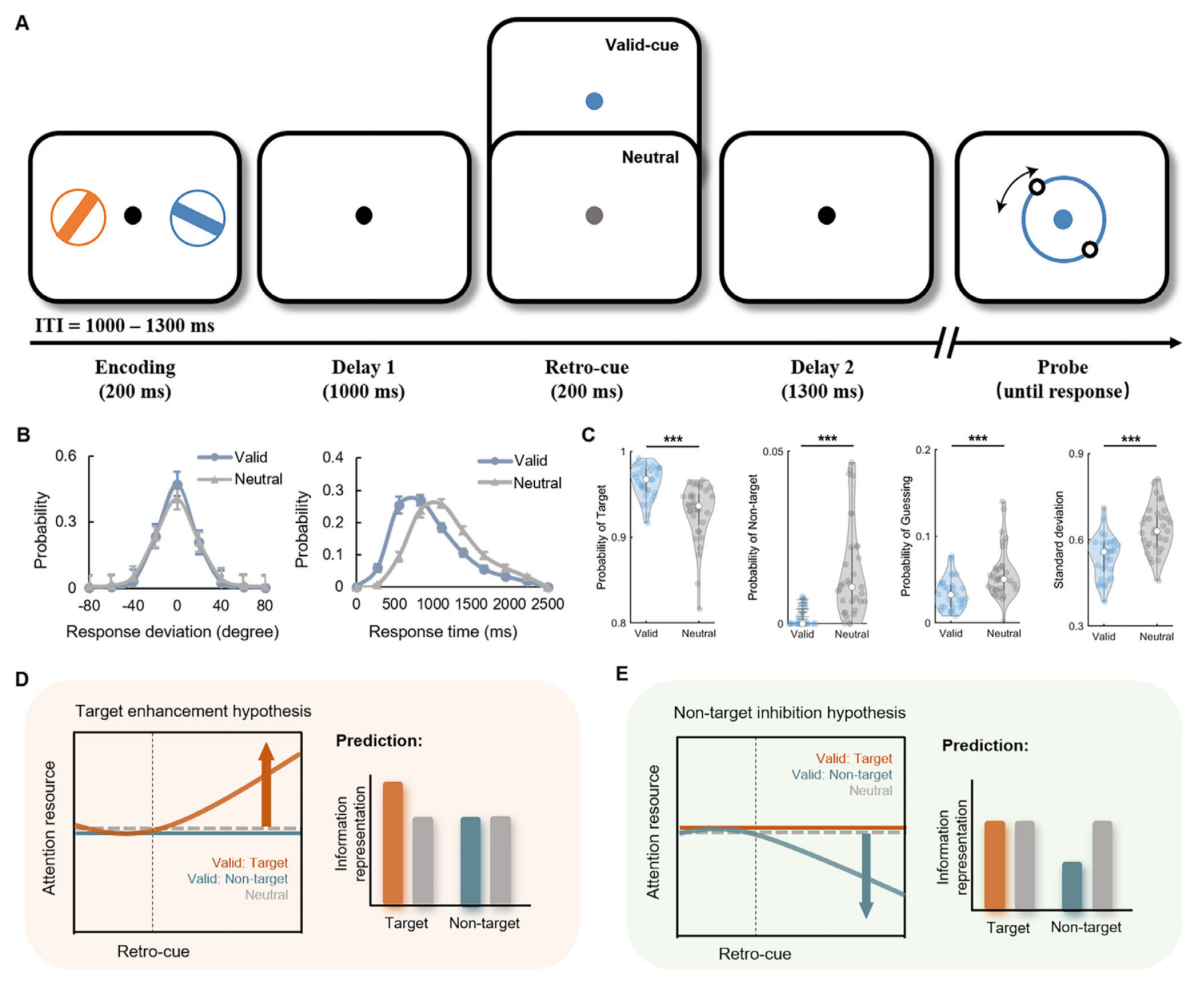
WM precision task and behavioral outcomes. (A) Trial sequences of the WM precision task with valid and neutral cues. (B) Probability of response deviation and response time in both the valid and neutral conditions. (C) Mixture modeling of the behavioral results. (D) Target enhancement hypothesis. We predicted a significant difference between valid retro-cue and neutral cues in the target orientation classification but no difference between valid retro-cue and neutral cues in the non-target orientation classification. (E) Non-target inhibition hypothesis. We predicted a significant difference between valid retro-cue and neutral cues in the non-target orientation classification but no difference between valid retro-cue and neutral cues in the target orientation classification. ITI, inter-trial interval; *** *p* < 0.001.

Here, we adopted a visual WM precision task with valid and neutral retro-cues (Fig. 1A) and recorded scalp electroencephalography (EEG) signals to directly examine the neurophysiological correlates of attentional effect on WM precision with both multivariate and univariate approaches. Multivariate decoding was applied to examine target enhancement (Fig. 1D) and non-target inhibition hypotheses (Fig. 1E). We further explored oscillatory correlates and connections to provide neurophysiological evidence of how retro-cues benefit WM representations.

## 2 Methods

### 2.1 Participants

Thirty-five healthy college students (18 females, age range: 19–28 years) participated in the EEG experiment. No statistical methods were used to previously determine sample sizes, but our sample size is similar to prior studies that applied a similar decoding analysis (Hajonides et al., 2021). All of them were right-handed and had normal or corrected-to-normal vision without color blindness. Two participants were removed due to excessive artifacts or ocular movement. Finally, thirty-three participants (17 females, age range: 20–28 years) were involved in further analysis. The current study was approved by the Beijing Normal University Institutional Review Board, and written informed consent was obtained from each participant.

### 2.2 Stimuli and procedures

Participants performed a retrospective covert attention task, which required them to select and attend to one of two visual representations maintained in WM. The stimuli were presented on a 21-inch LCD monitor (800 × 600 pixels, 60 Hz) through PsychoToolbox in the MATLAB (The MathWorks Inc., Natick, MA) environment with a viewing distance of 80 cm.

In the encoding stage, two bars surrounded by a ring (size: 4° × 0.32°) were presented at 5° from the left and right of fixation. Bars were randomly allocated to two different colors from blue (RGB: 21, 165, 234), orange (RGB: 234, 74, 21), green (RGB: 133, 194, 18), and purple (RGB: 197, 21, 234) and two different orientations from a set of eight: ±11.25°, ±33.75°, ±56.25°, ±78.75° The colors were independent across the bar locations and orientations.

Each trial began with an encoding display that contained two to-be-memorized bars (with different colors and orientations) for 200 ms, followed by a delay in which only a central fixation remained on the screen for 1000 ms. Then, a retrospective attentional cue was presented with a color-changed central fixation for 200 ms. In half of the trials, the color was randomly changed to one color of the two bars as a valid cue, indicating which bar needed to be recalled in advance. In the remaining trials, the color was changed to gray as a neutral cue, which provides no attentional benefits for a head start for retrieval. Trials were allocated to valid and neutral conditions randomly within each block. Then, followed by another 1300 ms delay after the retro-cue, a probe bar with a random orientation was displayed in the color of the cued bar (i.e., the target) in the valid-cue trials, while a probe was randomly presented in either color of the two bars in the neutral-cue trials. Participants were instructed to move the mouse and to press the left button to report the orientation of the target as precisely as possible within 4000 ms. After one practice block, the participants completed 20 blocks of 56 trials with a 1-min break between the blocks with EEG recording synchronously.

### 2.3 Behavioral modeling

The recall error distribution was first obtained from the angular distance between the reported and the actual orientation for each trial. Then, we fit the response error distribution with the mixture model (Bays et al., 2009) using the Analogue Report Toolbox in MATLAB. Maximum likelihood estimates of the parameters of the mixture model included the standard deviation (SD) and the probability of the target, non-target, and uniform responses. SD represents the width of the recall error distribution, indicating an inverse of WM precision. The probability of target, non-target, and uniform responses represents the probability of correctly reporting the target orientation, wrongly reporting the non-target orientation, and guessing a random orientation, respectively.

### 2.4 EEG recording and preprocessing

EEG signals were recorded using a cap with 64 AgCl electrodes in accordance with the international 10–20 system and a SynAmps EEG amplifier while performing the task. Eye movements and blinks were monitored using two electrodes placed 1 cm above and below the left eye for vertical electrooculogram (EOG) and another two electrodes placed at the outer canthus to the eyes for horizontal EOG. Before recording, electrode impedance was maintained below 5 kΩ. Signals were referenced online to the left mastoid, amplified with a bandpass of 0.01–400 Hz and digitized at a sampling rate of 1000 Hz.

Data preprocessing was performed using the EEGLAB toolbox (Delorme and Makeig, 2004) and custom scripts in MATLAB. EEG signals were first filtered with a bandpass of 0.1–40 Hz and offline referencing to the average of the two mastoids. Segments were then extracted from −2000 ms to 2500 ms relative to the retro-cue onset. Eyeblink and movement artifacts were corrected by independent component analysis (ICA). After ICA, the baseline correction was performed from −200 ms to retro-cue onset in each segment. Then, segments with voltages exceeding ±80 *μ*V at any electrode or exceeding ±50 *μ*V at the horizontal EOG electrode were also excluded. Last, two participants were removed from further analysis if more than 40% of segments were removed due to artifacts or the averaged horizontal EOG activity across the trials exceeded ± 3.2 *μ*V. These procedures removed 2.0% of trials (range: 0–10.5%) from the participants among the final samples, leaving the following number of trials (mean ± std) in each condition: 549±14 for the valid condition and 548±16 for the neutral condition.

### 2.5 Multivariate pattern classification

**Fig. 2:**
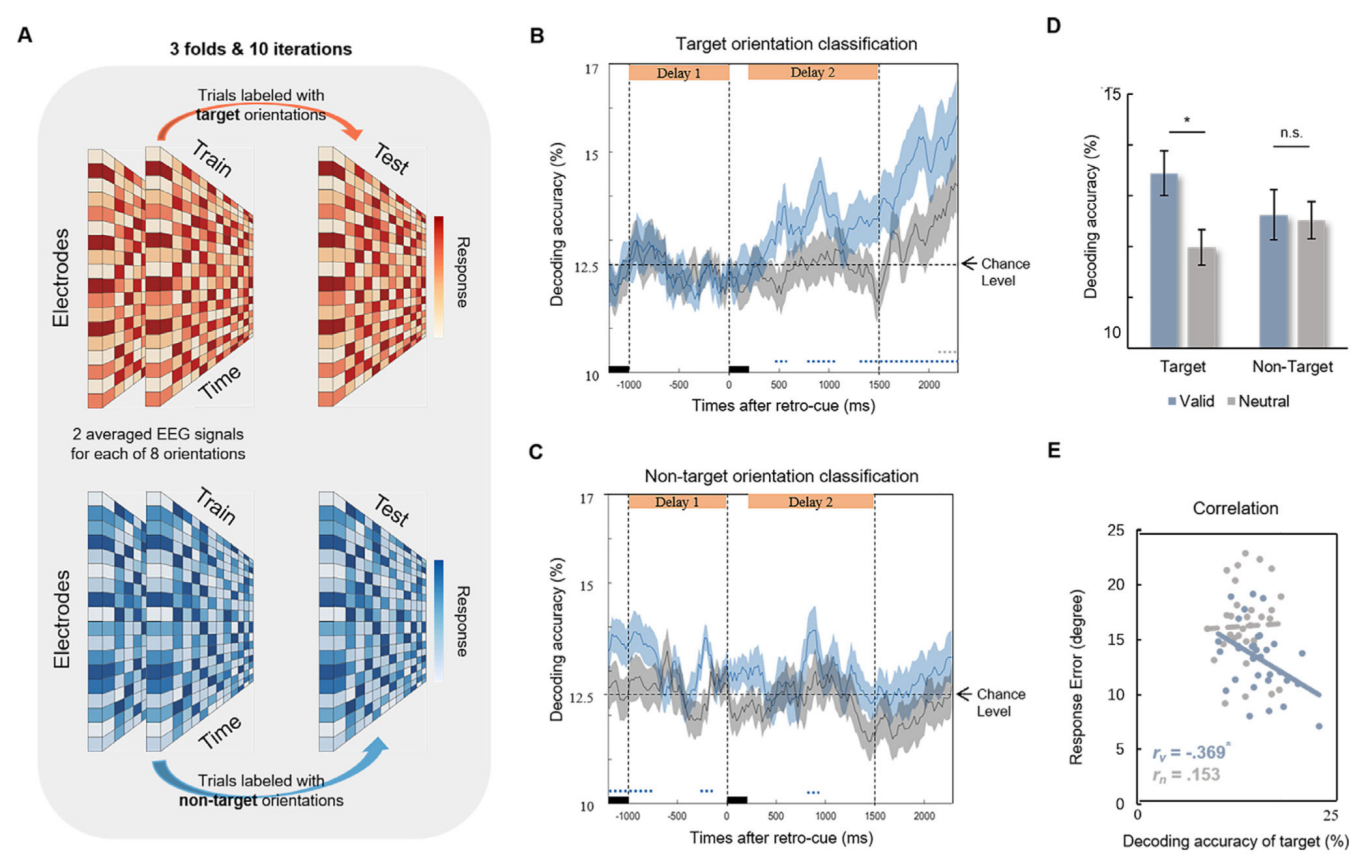
Orientation classifications. (A) Three-fold cross-validation was applied to trials labeled with target orientations (upper) and non-target orientations (below) to calculate the decoding accuracy of the multivariate pattern classification. (B) Temporal dynamics of the decoding accuracy for target orientation classification in the valid and neutral conditions. (C) Temporal dynamics of the decoding accuracy for non-target orientation classification in the valid and neutral conditions. (D) Averaged decoding accuracy of target and non-target orientation between 500 and 800 ms after the retro-cue supports the target enhancement hypothesis. (E) Individuals with higher decoding accuracy of the target orientation classification in the valid but not neutral conditions showed lower behavioral response errors. * *p* < 0.050; Blue and gray dotted lines represent significant time points where the decoding accuracy was higher than the chance level in valid and neutral conditions, respectively.

Multivariate pattern classification was applied to decode the orientations of the target and non-target objects (Fig. 2A). Each segment for decoding analysis was defined as −200 ms to 2400 ms around the onset of the retro-cue and was downsampled to 50 Hz to improve the stability of decoding by averaging several adjacent time points (Li et al., 2022a). Segments were labeled with different target/non-target orientations with 8 labels in total. Several segments with the same labels were arranged into three bins and averaged in each bin to improve the signal-to-noise ratio. The classifier was based on a linear support vector machine (SVM), and responses in all 60 electrodes serving as features were used to train classifiers through the MATLAB fitcecoc() function at each data point for each participant. The training and testing phases at a given data point were based on different segments. A three-fold cross-validation and ten iterations procedure was applied at each data point to minimize the fortuity caused by the trial assignments and to yield a more stable outcome (Bae and Luck, 2018; Adam et al., 2020; Hong et al., 2020). Decoding was considered correct only when the target/non-target orientation was determined correctly. Decoding accuracy was computed by comparing the true labels of the target/non-target orientations with the predicted labels, and the chance level was 12.5%.

The color and space of the target objects were also decoded through the same procedure. We calculated the temporal generation of color and space through decoding analysis (King and Dehaene, 2014). Specifically, classifiers were trained at a given time sample and evaluated for their ability to generalize to all of the time samples. Therefore, we could obtain a generalization map of decoding accuracy with the training times as the x-axis and the testing times as the y-axis. Our basic idea is that if we trained the classifier at a given time point (t_1_) and information could be decoded at another time point (t_2_), it means mnemonic representation was transformed from t_1_ to t_2_. However, if we trained the classifier at t_1_ but the information could not be decoded at t_2_, it means mnemonic representation was relatively transient across time. This approach allowed us to study the temporal dynamics of the color and space representations during WM. Our hypothesis is that if the mnemonic representational formats (EEG patterns) were similar between a given time point and the other time points, we would expect the classifier trained at that given time point could generalize to other time points. In other words, a higher than chance level off-diagonal decoding accuracy indicates that neural representations of color and space remain stable across time (Stokes, 2015). Therefore, we quantified the spread time for the decoding accuracy to identify the representation stability of color and space. The “S” was defined as an index to examine whether the decoding accuracy was higher than the chance level when the training and testing were based on different time points. The specific formulas are as follows:

$$Spd_{\left(t_{\text{train}},t_{test}\right)}=\{\begin{array}{c} 1,\;if\;ACC_{\left(t_{train},t_{\text{test}}\right)}>\;chance\;level\;\\ 0,\;if\;ACC_{\left(t_{train},t_{test}\right)}\leq \;chance\;level\; \end{array}$$

where ACC denotes the decoding accuracy of each training-testing paired time point from the temporal generation map. The chance level for color decoding is 25%, and for space decoding, it is 50%.

Then, to characterize the representation stability, we summed Spd up across the testing temporal dimension by using the following formula to obtain the spread time index over time.

$$Spread\;time_{\left(t_{train}\right)}=\sum_{t_{test}}Spd_{\left(t_{train},t_{test}\right)}$$

A larger spread time index represents the more testing time points where the color or space could share the same neural representations and be generalized from a given training time point, indicating a more stable feature representation.

To examine the significance of the decoding accuracy, segments were arranged to random labels to train and test the control classifiers. Then, a 10,000-time cluster-based permutation test was performed across the time (Bae and Luck, 2018).

### 2.6 Univariate analysis

For ERP analysis, each EEG segment was time-locked to the retro-cue onset (–200–2400 ms) and set to a baseline between –200 ms and 0 ms before the onset of retro-cue in each trial. The artifact-free EEG segments were then averaged for the valid condition and neutral condition separately. The frontal N600 was averaged between 600 ms and 1300 ms across 9 frontal-central electrodes (F1, Fz, F2, FC1, FCz, FC2, C1, Cz, C2), and the averaged frontal N2 was averaged between 320 ms and 360 ms across the same 9 frontal-central electrodes. Furthermore, we divided trials into three equal bins according to the behavioral RTs and then calculated the averaged frontal N2 in each RT bin.

For time-frequency analysis, EEG signals were segmented from –1800 ms to 2400 ms around the onset of the retro-cue. To obtain non-phase-locked spectral power, phase-locked ERPs were removed before any spectral measures were calculated to avoid oscillatory activities contaminated by ERPs (Wang and Ding, 2011). The instantaneous power and phase at a frequency range of 2–30 Hz in 0.5 Hz steps was estimated by applying a short-time Fourier transform to the Hanning-tapered data. Then, power was baseline-corrected between –1800 ms and –1400 ms (400 ms duration before the encoding onset) and transformed into Log space. Changes with respect to a baseline over time were referred to as ERD. Previous studies have reported that alpha power mainly occurs in the posterior cortex (Poch et al., 2022; Zhao et al., 2022a); therefore, alpha power (8–12 Hz) was estimated in all parieto-occipital electrodes (P3/4, PO3/4, PO5/6, P7/8, PO7/8, O1/2, Pz, Oz). Averaged posterior alpha power (8–12 Hz) in both valid and neutral conditions for statistics was then calculated between 300 and 600 ms after retro-cue (Zhao et al., 2022a).

The inter-trial phase coherence (ITPC) was calculated across trials through the newtimef function in EEGLAB, reflecting the consistency of phase values across trials within each electrode. The ITPC is stimulus-locked and independent of amplitude changes. The value of ITPC is between 0 and 1. A value closer to 0 indicates a lower phase synchronization across trials, and a value closer to 1 represents a higher phase synchronization across trials. Here, ITPC in the theta band (3–7 Hz) was calculated in the midline frontal electrode (Fz) and averaged between 100 ms and 700 ms to examine the difference between valid and neutral conditions.

To examine whether the retrospective attention indexed by posterior alpha amplitudes could be modulated by executive control indexed by frontal theta phases, we performed PAC analysis between frontal theta phases and posterior alpha amplitudes. We first extracted alpha amplitudes and theta phases by applying a Hilbert transform (hilbert.m) on filtered EEG signals. To acquire a stable estimation of PAC, we maximized the measurement window to the whole retention (0–1500 ms after retro-cue onset, > 6 theta cycles). The theta phases were calculated in the midline frontal electrode (Fz), and alpha amplitudes were calculated in all electrodes to examine connections between the mid-frontal cortex and the whole brain. Then, the PAC modulation index was calculated to assess the distribution of alpha amplitudes in different theta phases in both valid and neutral conditions (Tort et al., 2010). A larger PAC modulation index means a stronger coupling between two frequencies. The PAC index was then averaged across all parieto-occipital electrodes (same as electrodes used to estimate alpha power) to examine the difference between valid and neutral conditions.

## 3 Results

### 3.1 Behavioral benefits from retro-cue

Robust behavioral benefits were found after valid retro-cues (Fig. 1BC). Participants showed lower recall errors (*t*_*32*_ = 8.820, *p* <0.001, *d* = 3.118) and faster reaction times (RTs; t_32_ = 18.192, *p* <0.001, *d* = 6.432) in the valid condition. Then, maximum likelihood estimates were used to fit the response error distribution with the mixture model (see methods for more details). The modeling results also showed that valid retro-cues enhanced the probability of the target response (*t*_*32*_ = 6.444, *p* <0.001, *d* = 2.278), improved the recall precision (*t*_*32*_ = 11.790, *p* <0.001, *d* = 4.168), and reduced the probability of random guessing (*t*_*32*_ = 4.379, *p* <0.001, *d* = 1.548).

### 3.2 Target and non-target orientation decoding support the attentional strengthening hypothesis

To directly examine the information representations with two distinct hypotheses proposed in Fig. 1DE, multivariate pattern classification was applied to train the EEG data with different labels to identify whether the target and non-target orientations could be decoded from neural response distributions (Fig. 2A; see Methods). If the data support the target strengthening hypothesis, since the target representation is enhanced by valid retro-cue, we would expect a higher decoding accuracy of target orientation classification in the valid condition but no difference in decoding accuracy of non-target orientation classification. If the data support the non-target inhibition hypothesis, non-target representation will be inhibited by a valid retro-cue. Since rejection template formation of distractors could achieve decoding accuracy of distractor information above chance level (Wen et al., 2022), we would expect a higher decoding accuracy of non-target orientation classification in the valid condition but no difference in decoding accuracy of target orientation classification.

We found that valid retro-cues induced a higher decoding accuracy than neutral cues in target orientation classification (*t*_*32*_ = 2.388, *p* = 0.023, *d* = 0.844; Fig. 2B), but no difference was found in non-target orientation classification (*t*_*32*_ =0.086, *p* = 0.932, *d* = 0.030; Fig. 2C), supporting the target strengthening hypothesis (Fig. 2D). In the valid condition, the decoding accuracy of target orientation increased from delay 1 to delay 2 (*t*_*32*_ = 3.222, *p* = .003, *d* = 1.139; Fig. 2B), but no significant difference was found in the decoding accuracy of non-target orientation between delay 1 and delay 2 (*t*_*32*_ = 0.281, *p* = .781, *d* = 0.099; Fig. 2C). Importantly, individuals with higher decoding accuracy showed lower behavioral response errors in the valid condition (*r* = –.369, *p* = 0.035) but not in the neutral condition (*r* = 0.152, *p* = .397), indicating enhanced target representation by valid retro-cues (Fig. 2E).

### 3.3 Flexible temporal dynamic in color and space decoding

**Fig. 3:**
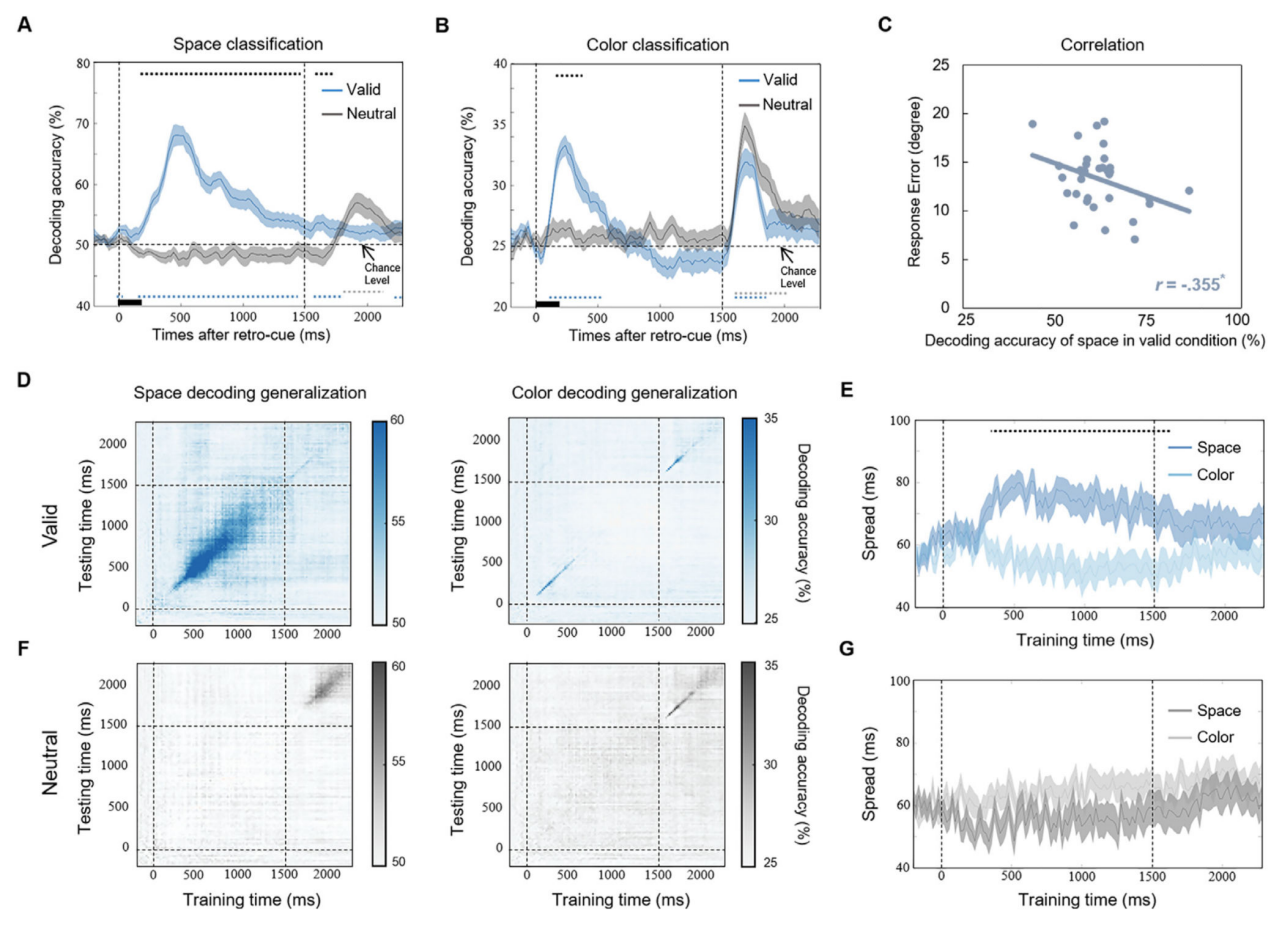
Target color and space classification. (A) Temporal dynamics of the decoding accuracy for target space classification in the valid and neutral conditions. (B) Temporal dynamics of the decoding accuracy for the target color classification in the valid and neutral conditions. (C) Individuals with higher space decoding accuracy in the valid condition showed lower behavioral response errors. (D) Temporal generalization of the decoding accuracy for the space and color classification of the target in the valid condition. (E) Temporal dynamics of spreading time for target color and space decoding accuracy in the valid condition. The black dotted line represents a time period with a significant difference. (F) Temporal generalization of the decoding accuracy for the space and color classification of the target in the neutral condition. (G) Temporal dynamics of spreading time for the target color and space decoding accuracy in the neutral condition. * *p* < 0.050. Blue and gray dotted lines represent significant time points where the decoding accuracy was higher than the chance level in valid and neutral conditions, respectively. Black dotted lines represent time points that showed significant differences between valid and neutral conditions.

We then decoded the space and color of the target to investigate whether features are binding to the goal-directed orientation to promote target representations. Both the color and space of the target could be decoded after retro-cue onset (Fig. 3AB), suggesting that the priority of the target was modulated by object-based attention, not just orientation-based attention. A repeated-measures ANOVA was performed with 2 cue types (valid, neutral) and 2 time periods (after retro-cue, after probe) as factors. Significant interactions were found in both the color (*F* = 23.896, *p* <0.001, *η*_*p*_^*2*^ = 0.428) and space (*F* = 50.534, *p* <0.001, *η*_*p*_^*2*^ =0.612) classifications, indicating the different decoding patterns of the valid and neutral conditions between the time after the retro-cue and after the probe. These results suggested that once the spatial information was retrieved after the retro-cue, it was not necessary to decode this spatial representation again after the probe. More importantly, a significant negative correlation was found between individual space decoding accuracy after the valid retro-cue and behavioral memory errors (*r* = –.355, *p* = 0.043; Fig. 3C), indicating that effective spatial binding could benefit WM representational precision. We also performed the decoding analysis on non-target color representations, and non-target color could not be decoded in either valid or neutral conditions (Fig. S1).

Temporal generalization analysis was conducted to investigate the representation dynamics of the different features (Fig. 3DF). This analysis used representations at one time point to decode the same feature in another time point, and a higher than chance level off-diagonal decoding accuracy means that neural representations remain stable across time (see Methods for more details). Space decoding generalization showed a larger temporal spread than color decoding generalization after valid retro-cues (*p*_*FDR-corrected*_ <0.05; Fig. 3E), but it did not show any difference after neutral cues (*p*_*FDR-corrected*_ >0.05; Fig. 3G), suggesting a stable representation of spatial information after valid retrospective attention.

### 3.4 Frontal ERP and theta phase track attentional control during WM

**Fig. 4:**
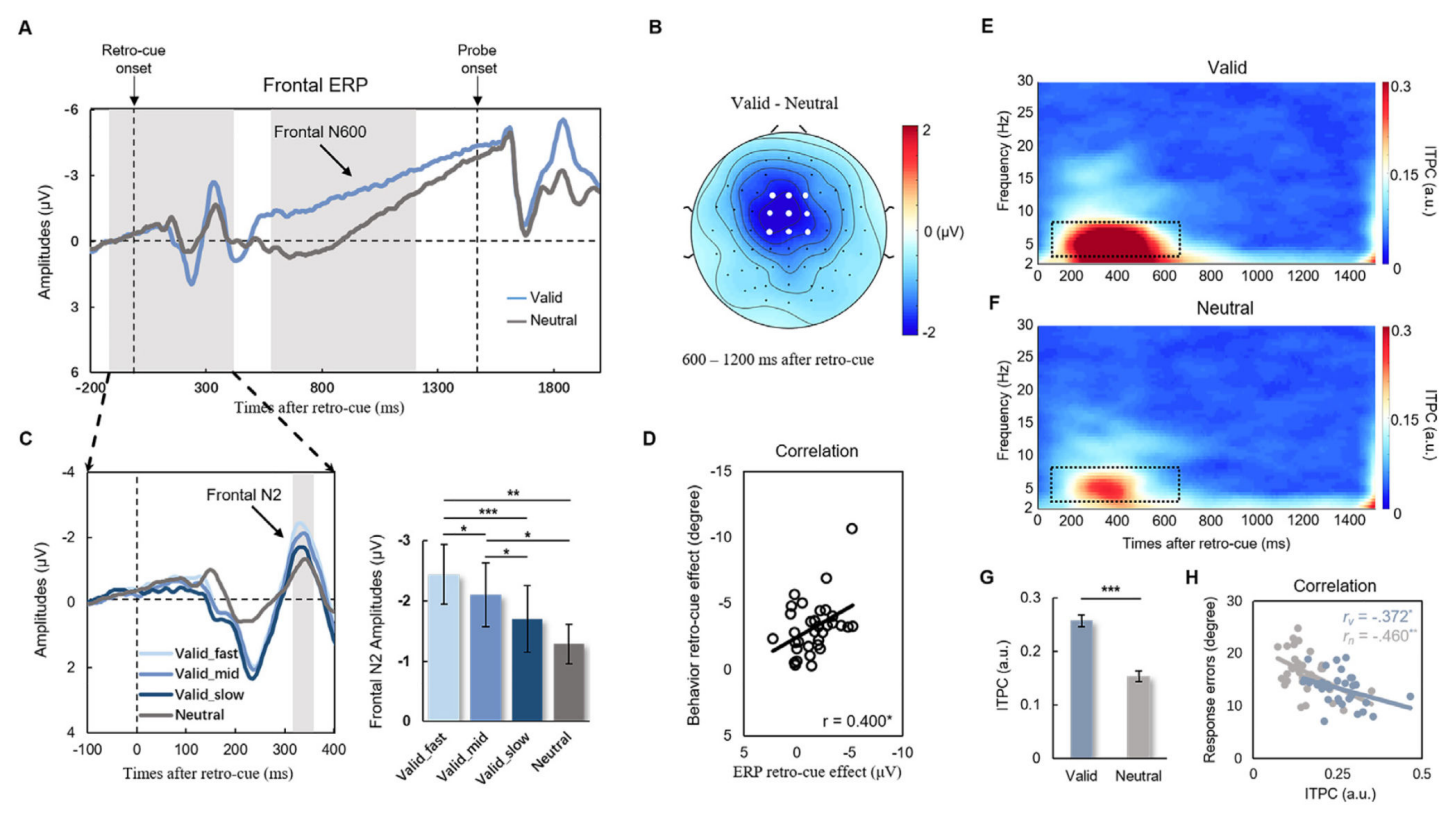
ERP and ITPC results. (A) Grand average waveforms of frontal ERPs for valid and neutral conditions. (B) An averaged topographic map (600–1200 ms) of the difference in frontal N600 amplitudes between valid and neutral conditions. White dots represent the electrodes used to calculate frontal N600. (C) Grand average waveforms of frontal ERPs for neutral and valid conditions with three different RTs. Frontal N2 amplitudes as a function of behavioral RTs across trials. (D) ERP retro-cue effect (frontal N600 in valid condition – frontal N600 in neutral condition) was correlated with behavioral retro-cue effect (recall errors in valid condition – recall errors in neutral condition). Time-frequency representations of ITPC in both valid (E) and neutral (F) conditions. (G) A higher theta ITPC averaged between 100 and 700 ms in midline frontal theta (3–7 Hz) was found in the valid condition than in the neutral condition. (H) Higher theta ITPCs predicted lower behavioral response errors in both valid and neutral conditions. * *p* < 0.05; ** *p* < 0.01; *** *p* < 0.001.

To investigate how neural activities contribute to retro-cue-related enhancement of target representations, we focused on the cue-evoked ERP component during the time with relatively high decoding accuracies of target orientation and spatial location. A large difference between valid and neutral conditions from 600 to 1200 ms (N600) was found in frontal-central electrodes. The topographic map of N600 is illustrated in Fig. 4B. Grand-average ERP waves across white dots marked frontal electrodes (Fig. 4A) showed a significant difference in averaged frontal N600 between valid and neutral conditions (*t*_*32*_ = 5.023, *p* <0.001, *d* = 1.776). Importantly, a significant correlation (*r* = 0.400, *p* = 0.021; Fig. 4D) was found between ERP retro-cue effect (difference in frontal N600 between valid and neutral conditions) and behavioral retro-cue effect (difference in recall errors between valid and neutral conditions), indicating that larger difference in frontal N600 between two conditions predicted larger behavioral benefits on WM precision across individuals. A marginally significant correlation between ERP retro-cue effect and target orientation decoding accuracy in the valid condition was further found (*r* = 0.336, *p* = 0.056), which might link the ERP component with decoding accuracy and partly explain how retrospective attention enhances representational precision in WM. Furthermore, when we divided trials into three equal bins according to the behavioral RTs (Fig. 4C), a repeated-measures ANOVA on frontal N2 amplitudes showed a significant main effect on RT (*F* = 6.981, *p* <0.001, *η*_*p*_^*2*^ =0.179), indicating frontal N2 as a function of RTs across trials. These results suggested that retro-cue evoked frontal ERP could track both precision (N600) and speed (N2) benefits of WM behavioral outcomes.

To some extent, the ERP wave is related to the phase-locked characteristic of oscillatory activities. Exploring the oscillatory mechanism of retro-cue benefits could help us understand the frequency contributions to the abovementioned frontal ERP components (Fig. 4EF). We found that inter-trial phase coherence (ITPC) in midline frontal theta (3–7 Hz) in the valid condition was significantly higher than that in the neutral condition (*t*_*32*_ = 11.687, *p* <0.001, *d* = 4.132; Fig. 4G), suggesting better information integration and cognitive control in the prefrontal cortex after valid retrospective attention. More importantly, significant correlations were found between theta ITPC and response errors in both valid (*r* = –.372, *p* = 0.033) and neutral (*r* = –.460, *p* = 0.007) conditions, indicating the important role of ITPC in target representation with stronger theta phase coherence leading to precise representation and lower behavioral response errors.

### 3.5 Alpha power and long-range connection within the frontoparietal network

**Fig. 5:**
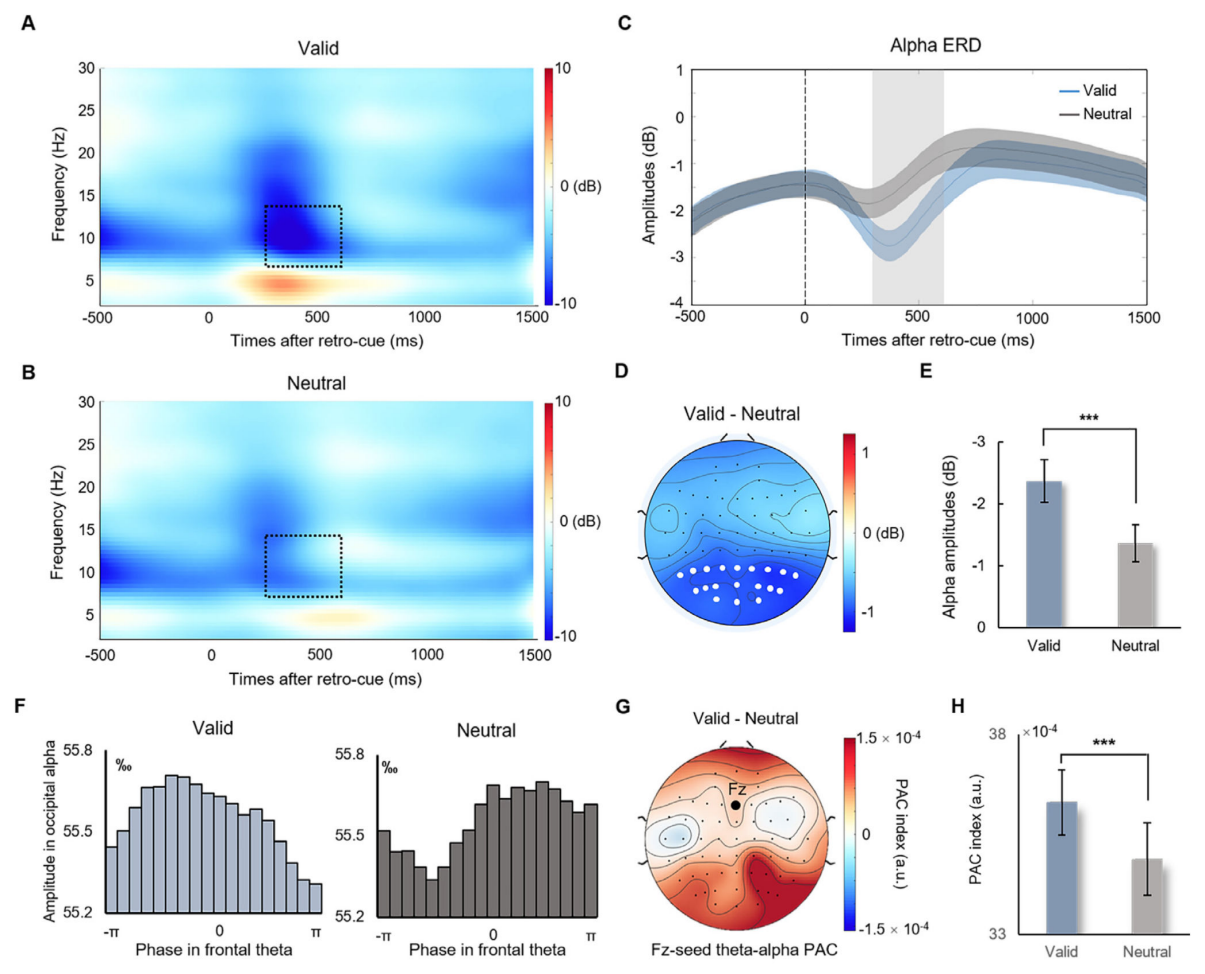
Oscillatory results. Time-frequency representations in valid (A) and neutral (B) conditions. Black dashed squares represent alpha power (8–12 Hz) between 300 and 600 ms. (C) A larger alpha ERD was found in the valid condition than in the neutral condition. The gray area represents the duration (300–600 ms) used to calculate the averaged alpha power. (D) An averaged topographic map (300–600 ms) of the difference in alpha power between valid and neutral conditions. White dots represent the electrodes used to calculate alpha power. (E) Averaged alpha power in valid and neutral conditions showed a significant difference. (F) The alpha amplitudes across different theta phase bins in valid and neutral conditions. (G) A topographic map of the difference between valid and neutral conditions in theta-alpha PAC between Fz and all electrodes. (H) The PAC index calculated between Fz and all parieto-occipital electrodes was larger in the valid condition than in the neutral condition. *** represents *p* < 0.001.

To examine how valid retro-cues modulated spatial attention to enhance target representation, we calculated ERD in alpha band (8–12 Hz) oscillatory activities during the retention of visual WM. As illustrated in Fig. 5AB, a significant ERD in alpha power over parieto-occipital sensors was found after retro-cues in time frequency representations of both valid (*t*_*32*_ = –6.775, *p* <0.001, *d* = 2.395) and neutral (*t*_*32*_ = –4.398, *p* <0.001, *d* = 1.555) conditions. The alpha ERD was larger in the valid condition than in the neutral condition (*t*_*32*_ = –5.606, *p* <0.001, *d* = 1.982; Fig. 5CE), and the corresponding topographic map of the difference in alpha power between the valid and neutral conditions is shown in Fig. 5D. This result suggested that more attention resources were deployed after the valid retro-cue during retention.

Since the retro-cue could enhance target representations by modulating both frontal theta phase and posterior alpha power, we then investigated how these modulations were binding to the target by examining the frontal-posterior long-range connection through theta-alpha PAC (see Methods). The alpha amplitudes across different theta phase bins (Fig. 5F) showed a curved uneven distribution in the valid condition but a relatively even distribution in the neutral condition. A topographic map of the difference between valid and neutral conditions in theta-alpha PAC between Fz and all electrodes is shown in Fig. 5G. We found a stronger theta-alpha PAC between Fz and all parieto-occipital electrodes in the valid condition than in the neutral condition (*t*_*32*_ = 4.818, *p* <0.001, *d* = 1.703; Fig. 5H). Importantly, a significant correlation (*r* = –.372, *p* = 0.033) was found between Δtheta-alpha PAC (difference between valid condition and neutral condition) and Δprobability of guessing, suggesting that a larger retro-cue effect in theta-alpha PAC predicted lower behavioral guessing rates. These results indicated that frontoparietal connection might be a fundamental mechanism of retrospective attention during WM retention.

## 4 Discussion

How attention prioritizes relevant information held in WM to support WM flexibility is the foundation of how the brain resolves the limit of WM resources. In the present study, we applied both multivariate and univariate analyses to investigate whether and how retrospective attention improved WM performance with a typical retro-cue WM task. Only representations of targets were enhanced and prioritized by retro-cues, which supports the target strengthening hypothesis. The task-irrelevant feature of the target, spatial location, could be decoded with high priority in the valid condition, indicating that spatial-object binding is vital to WM representation precision. Furthermore, mid-frontal theta ITPC predicted individual differences in WM precision across participants, and N2 components tracked moment-to-moment fluctuations in reaction time within participants. In addition, spatial attention-related parietal alpha ERD and its coupling with mid-frontal theta phase were modulated by valid retro-cues, further emphasizing neural correlates and connections within frontoparietal attentional networks.

### 4.1 Valid retrospective attentional modulation strengthens target representations

The most vital contribution of the present study is to provide new neurophysiological evidence for an attentional enhancement model of retrospective cues. Extensive behavioral and neural evidence has shown that retro-cues modulate attention in working memory (Gunseli et al., 2015; Poch et al., 2017); however, whether this attention reflects target enhancement or non-target inhibition is still under debate. Recently, an increasing number of studies have pointed out that attentional selection and non-target inhibition are not two sides of the same coin but instead reflect distinct neural mechanisms (Wöstmann et al., 2019; Zhao et al., 2022b). Consistent with previous studies (Makovski and Pertzov, 2015; Shimi and Scerif, 2022), we used a mixture model to fit behavioral responses and revealed that retrospective attentional cues can enhance target response probability. More importantly, EEG-based multivariate pattern classification analysis showed that the decoding accuracy of target orientation started to be higher than chance level about 500 ms after the valid retro-cue favoring the target strengthening hypothesis. The decoding accuracy of the target and the behavioral cueing benefits are correlated across individuals. However, the decoding accuracy of non-target orientation after the valid retro-cue kept fluctuating around chance level during the whole retention, not favoring the non-target inhibition hypothesis. The absence of non-target inhibition might be due to the memory load in our task being low, as previous work found that more non-targets were removed from WM with increasing memory loads (Van Moorselaar et al., 2015). Testing the non-target inhibition hypothesis with high-load WM tasks might be helpful in future studies.

In the neutral condition, the observation that the decoding accuracies of both target and non-target orientations were at chance level suggests that both target and non-target representations are held in activity-silent WM depending on short-term synaptic plasticity (Stokes, 2015; Wolff et al., 2017). Two similar orientations in the memory space have a close distribution of neuronal coding patterns, which explains why either target or non-target could not be decoded in the neutral condition. The successful decoding of the target in the valid condition might be because the valid retro-cue pings the brain to activate the target representation from the hidden states during retention. Retrospective attentional cues might provide another cluster of neurons involved in the mnemonic representation of the target, thereby amplifying the difference in the mnemonic representation of the target and non-target. Together, these results supported the hypothesis that the target representation was enhanced by the retrospective attentional cue during visual WM.

### 4.2 Flexible feature representations interact with retrospective attention in visual WM

Previous studies have shown that internal attention can prioritize a subset of mnemonic representations (Griffin and Nobre, 2003; Sheldon & Postle, 2020). If the mnemonic information was stored in the form of feature-based information, we would expect only task-relevant features could be decoded. However, if the mnemonic content was stored in the form of object-based information, we would expect both task-relevant and task-irrelevant features could be decoded. In our study, both task-relevant features (orientation and color) and the task-irrelevant feature (space) could be decoded after valid retro-cues. This result might support the integration hypothesis that mnemonic information was stored as discrete mnemonic objects in WM (Luck and Vogel, 1997), which could be modulated by retro-cue.

The flexibility of WM representations suggested that we can hold anything in mind with various neural representations (Buschman, 2021). Although retrospective cues can enhance the representation of target objects, different features exhibit different temporal dynamics, which might reflect flexible representations of different features held in WM (Bouchacourt and Buschman, 2019). For color representation during retention and retrieval, a higher decoding accuracy after retro-cue than after probe in valid conditions suggested that effective retrospective attentional cues activate memory representations of task-relevant visual objects through color. The encoding of the target space appears to exhibit different neural dynamics compared with the color encoding. Our findings indicate that even though the probe is presented in the center of the screen, the task-irrelevant target peripheral location can still be represented in neural activities. Recent micro-saccade studies reported that after visiting the location after the retro-cue, unless you did not pay attention during retention, you did not need to return after the probe (Draschkow et al., 2022; van Ede et al., 2019b). Our observations provide further neurophysiological evidence for the findings of eye movements; specifically, although the spatial location is task-irrelevant, retrospective attentional cues can continuously focus attention on the target side, helping to activate and retrieve object-based target representations in WM in advance. Additionally, computational models showed that persistent neural activities could be represented by attractor dynamics (Durstewitz et al., 2000; Wimmer et al., 2014; Wang, 2001). Temporal generation of the target space showed that valid retro-cues induced a more stable representation of space than color, indicating that valid retro-cues modulated spatial attention through persistent neural activities. These findings suggested that highly dynamic representations and integration of different features might be good candidates to organize the flexibility of WM representations. Further work is necessary to examine the temporal dynamics of space and color representations with equal probability.

Furthermore, the onset latency of space decoding accuracy is consistent with previous ERP findings in spatial selective attention (Eimer, 2014; Li et al., 2021, 2022b). Traditional covert attention contains a physically salient stimulus, which might confuse bottom-up attention capture with top-down attentional selection. Here, we used meaningful color as the retro-cue to isolate top-down control of objects held in WM, indicating object-based attentional modulation in visual WM. Location has been found to be binding to letter automatically (Campo et al., 2010; Elsley and Parmentier 2015). Previous studies have claimed that low WM precision is related to spatial binding errors (Ma et al., 2014; Pertzov et al., 2012). Our object-location binding results extending these findings by linking space decoding accuracy with behavioral precision. Specifically, a negative correlation between space decoding accuracy and response errors revealed that spatial binding of the target object benefited working memory precision. We propose that retrospective spatial attention optimized object-location binding and benefited WM precision. Our observations suggested that object-based representation can be enhanced by retrospective attentional cues for upcoming behavioral performance.

### 4.3 Mid-frontal neural activities track trait- and state-level variability in attentional preparatory processes during visual WM

We found a significant mid-frontal theta ITPC in both conditions, and it was correlated with individual behavioral recall errors in each condition, suggesting the critical role of ITPC in representing targets in both conditions. These results are consistent with previous results that the midline theta activity in the frontal area is related to cognitive control (Itthipuripat et al., 2013) and that theta phase has been thought to be associated with memory encoding (Berger et al., 2019). We also found a higher ITPC in the valid condition, which extended previous observations that the theta phase in the midline prefrontal cortex (PFC) coordinates retrospective attention through brain clocking mechanisms and enhances target representation held in WM.

Corresponding to the higher frontal theta ITPC in the valid condition we observed, we also found increased frontal N2 evoked by valid retrospective attentional cues. More importantly, the amplitudes of frontal N2 tracked the response speed of WM within individuals, indicating moment-to-moment fluctuations in attentional control and favoring the ‘readiness to remember’ (R2R) framework (Madore and Wagner, 2022). This R2R framework explains trait- and state-level variability in mnemonic performance as a function of preparatory attention and goal coding and their interactions with core mnemonic representations. Our findings suggested theta phase coherence not only as a neural marker of behavioral individual differences (theta ITPC) but also of real-time top-down attentional fluctuations over time (N2), which provides direct neural evidence that both within- and between-individual variability in attentional preparatory processes influences goal-directed behavior.

We further found that the fronto-central ERP component N600 differed between valid and neutral conditions and that the difference in N600 amplitudes between the two conditions contributed significantly to behavioral retro-cue effects. According to the positive correlation between the retro-cue effect on N600 and the retro-cue effect on behavioral error, our results might suggest a neural marker tracked behavioral benefits from retrospective attention on WM representations. Further work is needed to investigate and replicate the underlying neuronal mechanism of attentional modulation during WM retention.

### 4.4 Alpha oscillation gates the feed-forward information flow to integrate and enhance WM representations

Alpha oscillatory activity has been thought to index the deployment of visual attention (Klimesch, 2012). Whether alpha oscillations are casually linked to visuospatial attention is still under debate (Peylo et al., 2021). Antonov et al. (2020) suggested that modulation in alpha amplitudes is likely to be a consequence rather than a causal substrate of attention shifts. However, previous studies applying alpha-related noninvasive brain stimulation provided some convincing evidence for the causal link between alpha activity and visuospatial attention (Borghini et al., 2018; Kasten et al., 2020; Kemmerer et al., 2022a, 2022b; Romei et al., 2010). Although it is clear that alpha oscillations were related to the non-target inhibition (Gutteling et al., 2022; Jensen and Mazaheri, 2010), it is debated whether the inhibition by alpha oscillations is under direct top-down control. Other work has linked alpha oscillations with target enhancement during visual selective attention (Foster and Awh, 2019; Noonan et al., 2016). Further work is needed to characterize the causal role of alpha oscillations in selective attention.

Visuospatial attention does not solely modulate alpha amplitude but also the connection within frontoparietal networks (Fiebelkorn et al., 2018). Recently, more direct evidence was provided to support the role of alpha oscillations in feature integration (Ghiani et al., 2021; Parto Dezfouli et al., 2021; Zhang et al., 2019). Confirming the enhanced representation of the target space by valid retro-cues in multivariate pattern analysis, we also found larger alpha lateralization over the parieto-occipital cortex after valid retro-cues (Fig. S2), which is consistent with previous studies (Fu et al., 2022; Poch et al., 2017; Schneider et al., 2015). Our findings indicated that alpha power shaped the information flow held in WM and modulated the priority of the cued object gated by enhancing spatial attention representation and binding different features (Zhigalov and Jensen, 2020).

### 4.5 Organizing retrospective attention in visual WM through clocking mechanisms of theta-alpha phase-amplitude coupling in the frontoparietal connection

Consistent with previous findings in fMRI studies (Sprague et al., 2016), our EEG results provided direct neural evidence for WM representations being enhanced by retrospective attention and furthermore provided insight on the oscillatory mechanisms underlying attentional enhancement. Previous studies have reported the vital role of the fron-toparietal network in top-down attentional control (Hopfinger et al., 2000; Wang et al., 2010). We found that coupling of theta phases in midline PFC and parietal alpha amplitudes during WM retention was modulated by valid retro-cues, which extends neurophysiological evidence for the engagement of frontoparietal attentional networks in visual WM. This PAC induced by valid retrospective attention was different from many previous studies that found a local PAC in the same brain area (Daume et al., 2017) because our PAC result was based on a long-range connection between frontal theta phase and parietal alpha amplitudes. We suggest that posterior alpha oscillation would be nested within frontal slow fluctuation of excitability (theta-band) during retention, which would thus be involved in organizing object processing over time. The theta oscillations originating from frontoparietal networks, under attentional control, serve as a brain clock to organize the attentional states after the retro-cue (Fiebelkorn et al., 2018; Gaillard and Ben Hamed, 2022; Gregoriou et al., 2009; Helfrich et al., 2018). Importantly, the increased retro-cue effect on theta-alpha PAC predicted the reduced guessing rate. This long-range frontoparietal connection controlled by retrospective attention supported the fluctuations of top-down information flow in the engagement of frontoparietal networks (Madore and Wagner, 2022), which could guide goal-directed WM performance.

Convincing evidence was provided that the behavioral sampling of internal visual working memory representations was at a theta rhythm (Chota et al., 2022). Neural oscillations in the frontoparietal network modulate attention and perceptual sensitivity. A previous study found a PAC between frontal theta and parietal alpha in local field potentials when non-human primates performed spatial attention tasks (Fiebelkorn et al., 2018). Our findings, suggesting that alpha power in posterior areas might integrate perceptual features into discrete mnemonic objects (Luck and Vogel, 1997), might provide potential neural mechanisms for attentional sampling during the retention of working memory. A recent opinion proposed that rhythmic attention is a basis of cognitive flexibility (Fiebelkorn and Kastner, 2019). The flexible WM model predicting an increasing interaction between prefrontal and sensory regions (Buschman, 2021) was supported by the PAC results between prefrontal and posterior areas. Due to the highly flexible representation in WM (Bouchacourt and Buschman, 2019; Lorenc et al., 2018), our findings supported that the interaction between retrospective attention and flexible mnemonic representation could enhance and prioritize target representation held in WM.

### 4.6 Conclusions

With a typical retro-cue WM task, we provided convincing neuro-physiological evidence supporting object-based attentional strengthening theory. Neural correlates and connections within frontoparietal networks track retro-cue benefits on working memory precision. Retrospective spatial attention optimized object-location binding and benefited WM precision by prioritizing target representations. Our observations provide new insight into training individuals who suffer from memory deficits by modulating attention.

## Supplementary Material

Supplementary material associated with this article can be found, in the online version, at 10.1016/j.neuroimage.2023.119902.

Supplementary Material

## Data Availability

We have deposited the behavioral and EEG data in the present study on figshare (https://figshare.com/projects/Li_et_al_Neuroimage_2023_RetrospectiveWM/158117). Custom codes for data analysis are available on github (https://github.com/DongweiLi1/Codes-of-Li-et-al.-Neuroimage-2023). Any additional information that supports the findings of this study will be available from the corresponding author upon request.

## Data availability

Data will be made available on request.
