# Supplementary material for: Prioritizing flexible working memory representations through retrospective attentional strengthening
Source: Neuroimage. Author manuscript; Available in PMC 2025 Sep 18. (PMC7618137; doi:10.1016/j.neuroimage.2023.119902)
Supplement: Supplementary Material [file EMS208343-supplement-Supplementary_Material.docx]

**Supplementary Information**

**Prioritizing flexible working memory representations through retrospective attentional strengthening**

Dongwei Li^a,b,1^, Yiqing Hu^a,1^, Mengdi Qi^a^, Chenguang Zhao^a,d^, Ole Jensen^b^, Jing Huang^d,*^, Yan Song^a,c,*^

^a^ State Key Laboratory of Cognitive Neuroscience and Learning & IDG/McGovern Institute for Brain Research, Beijing Normal University, Beijing, China

^b^ Centre for Human Brain Health, University of Birmingham, Birmingham, UK

^c^ Center for Collaboration and Innovation in Brain and Learning Sciences, Beijing Normal University, Beijing, China

^d^ Center for Cognition and Neuroergonomics, State Key Laboratory of Cognitive Neuroscience and Learning, Beijing Normal University, Zhuhai, China

^1^ These authors contributed equally

^*^ Correspondence: Jing Huang: huangjing_zhu@bnu.edu.cn; Yan Song: songyan@bnu.edu.cn

**List of Contents**

1. Non-target color classification.

2. Alpha lateralization in the valid condition.

3. Additional correlation analysis.

Figures S1.

Figures S2.

Figures S3.

**1. Non-target color classification.**

Similar to the non-target orientation classification analysis (**Fig. 2C**), we also examined the decoding accuracy of the non-target color. Results showed that no significant difference was found in the decoding accuracy of the non-target color between valid and neutral conditions (*p_corrected_* >.05; **Fig. S1**). This result provided extended neural evidence that retro-cue benefits on color encoding did not follow the non-target inhibition hypothesis.


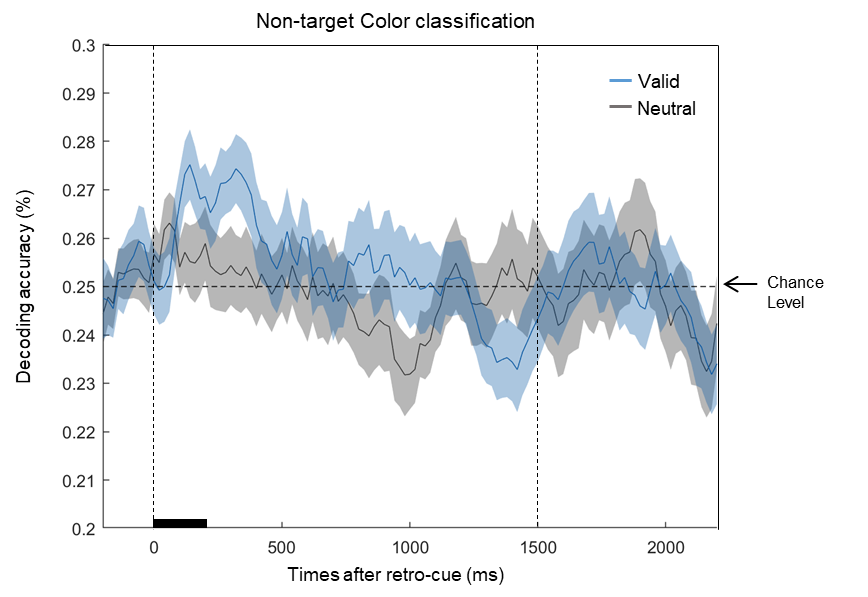


**Fig. S1.** Temporal dynamics of the decoding accuracy for the non-target color classification in the valid and neutral conditions.

**2. Alpha lateralization in the valid condition.**

Respective attention can induce a more negative target-contralateral alpha power, compared with target-ipsilateral alpha power during retention (Poch et al., 2017). We also find a similar alpha lateralization (calculated by alpha power contralateral to the target minus alpha power ipsilateral to the target) after the valid retro-cue (*t_32_* = 2.277, *p* = .030, *d* = .805; **Fig. S2**).

Consistent with previous studies (Mössing & Busch, 2020), we did not find a significant correlation between alpha lateralization and response errors (*r* = –.014; *p* = .94). However, we did find a marginally significant correlation between alpha lateralization and behavioral target response rate (*r* = –.298; *p* = .092), but no significant correlation was found between alpha lateralization and non-target response rates (*r* = 066; *p* = .716). These correlation results to some extent suggested that alpha lateralization might be related to target enhancement rather than distractor inhibition.


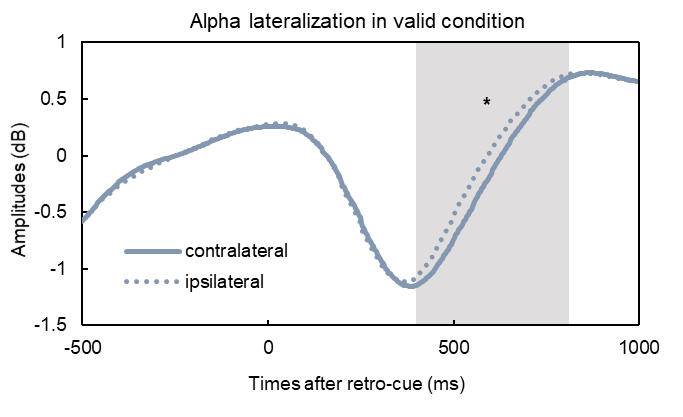


**Fig. S2.** Alpha power was more negative in the target-contralateral electrodes than in the target-ipsilateral electrodes in the valid condition. The gray area represents the duration (400–800 ms) used to calculate the averaged alpha power. * represents *p* <.05.

**3. Additional correlation analysis.**

We have calculated the correlations among several neural correlates (including decoding accuracy, ERP, ITPC, and PAC). Results showed a significant positive correlation between the theta ITPC and decoding accuracy for target color in the valid condition (*r* = .550, *p* < .001; **Fig. S3**), indicating that the frontal theta phase might be involved in the retrospective attention by coordinating target color. Decoding results are blind-sourced and thus always lack neurophysiological evidence. The significant correlation between decoding results and the oscillatory signals suggested the potential neurophysiological relevance of the machine learning approach in mnemonic representations. Another marginally significant correlation was found between alpha ERD and theta-alpha PAC (*r* = .306, *p* = .083). No other significant correlation was found (*ps* > .107).

Additional correlations between several neural correlates and behavioral indexes (target response rate, non-target response rate, guessing rate, standard deviation, and response time). Results showed a significant negative correlation between the decoding accuracy for target space and the standard deviation in the valid condition (*r* = –.382, *p* = .028), indicating that higher spatial representation of the target could predict lower working memory precision. Another marginally significant correlation was found between theta ITPC and non-target response rate in the valid condition (*r* = –.323, *p* = .067). No other significant correlation was found (*ps* > .115).


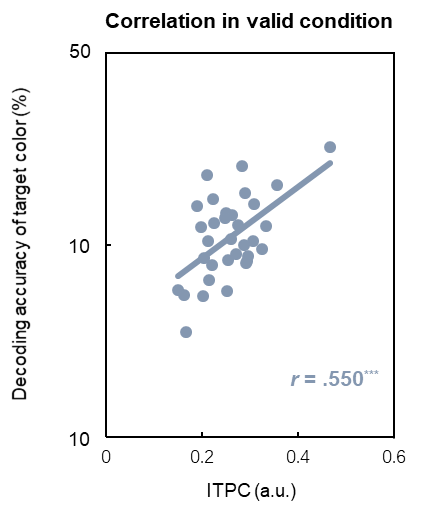


**Fig. S3.** A higher decoding accuracy of the target color was correlated with a higher theta ITPC in the valid condition.

**References**

Poch, C., Capilla, A., Hinojosa, J.A., Campo, P., 2017. Selection within working memory based on a color retro-cue modulates alpha oscillations. Neuropsychologia 106, 133-137.

Mössing, W.A., Busch, N.A., 2020. Lateralized alpha oscillations are irrelevant for the behavioral retro-cueing benefit in visual working memory. PeerJ 8, e9398.
